# Book Review: Data Collection Research Methods in Applied Linguistics

**DOI:** 10.3389/fpsyg.2021.668712

**Published:** 2021-03-24

**Authors:** Yu Zhang

**Affiliations:** School of Foreign Languages, Northeast Normal University, Jilin, China

**Keywords:** data collection, applied linguistics, qualitative research method, quantitative research method, mixed-method approach

Choosing the appropriate data collection methods is the key to obtaining reliable and valid research data. *Data Collection Research Methods in Applied Linguistics* highlights the importance of data collection, presents a variety of approaches for obtaining data and provides practical guidance for applied linguistics researchers with ample examples from published articles and books.

This book contains 12 chapters. Chapter 1 briefly introduces various research designs, including experiments, surveys, and case studies in applied linguistics. Chapters 2 to 9, which form the main body of the book, describe direct and indirect data collection methods. Chapters 2 to 5 analyze direct ways of acquiring data on participants, including language elicitation tasks (Chapter 2), introspective and retrospective tasks (Chapter 3), tests (Chapter 4), and observations (Chapter 5). Chapters 6 to 9 consider indirect ways of collecting data on participants through self-reporting, including interviews (Chapter 6), diaries, journals, and logs (Chapter 7), questionnaires (Chapter 8), and focus groups (Chapter 9). Chapters 10 and 11 outline direct and indirect techniques for obtaining spoken and written discourses to construct and use corpora, such as the British National Corpus. Finally, Chapter 12 discusses how to improve data validity and reliability by using triangulation and how to ensure research transparency, which allows future researchers to replicate studies.

This book makes itself distinctive from other research methodology books in terms of its explicit focus on data collection, unique categorization of data collection methods, and structural components promoting reader interactions. First, this book primarily presents data collection methods without associating them with particular research designs. This explicit focus on data collection creates more space to feature a variety of approaches, including widely used methods, such as questionnaires, and less commonly used methods, such as logs and focus groups. Thus, this book can encourage researchers to flexibly and creatively integrate various data collection methods within a research design.

Second, this book categorizes data collection methods based on whether the data are obtained from participants directly, such as through role playing and storytelling, or indirectly, such as through written interviews. Compared with other research methodology books, such as those that classify data collection methods according to whether the data are primary or secondary (e.g., Kothari, 2004) or quantitative or qualitative (e.g., Dörnyei, 2007), this new classification approach aims to “provide guidance in this area by squarely focusing on the things researchers do to obtain data in their research projects” (Rose et al., 2020, p. vii).

Third, this book's structural arrangement includes reflective activities and instructive examples to foster reader interactions. Each chapter starts with pre-reading activities, which involve thinking, discussing, and imagining, and ends with post-reading activities, which involve reflecting, expanding, and applying, in order to provoke contemplation and further discussion. Each chapter also explains key concepts and provides specific ways to improve data reliability and validity. For example, Chapter 8 introduces many strategies to reduce bias when constructing questionnaire, such as writing concise items, using simple language, avoiding negative and multiple choice questions, and considering the order effect and the response rate. To provide readers with practical guidance, data collection methods are analyzed using instructive examples from published studies, which involve not only quantitative and qualitative designs but also mixed-method designs. For instance, Chapter 9 contains six examples of studies that employed focus groups as a data collection method in different research designs, including quasi-experimental and mixed-method research.

Meanwhile, some aspects of this book could benefit from further development in future editions. For instance, Chapter 5 introduces many observation instruments, such as the Motivation Orientation of Language Teaching (Guilloteaux and Dörnyei, 2008); however, the brief explanations may make it difficult for novice researcher to apply these instruments effectively. Novice researchers would benefit from the inclusion of complete observation sheets and more detailed depictions of what to observe and how to record observations.

In summary, novice researchers and postgraduate students will find this book as an essential reference as they embark on their research journeys in applied linguistics. By introducing a variety of data collection methods, this book can inspire researchers and students to creatively adopt different approaches to obtain data when conducting research, such as using questionnaires and observations in a case study.

## Author Contributions

The author confirms being the sole contributor of this work and has approved it for publication.

## Conflict of Interest

The author declares that the research was conducted in the absence of any commercial or financial relationships that could be construed as a potential conflict of interest.
